# Water treatment at the point-of-use and treatment preferences among households in Ethiopia: A contemporaneous systematic review and meta-analysis

**DOI:** 10.1371/journal.pone.0276186

**Published:** 2022-10-27

**Authors:** Aiggan Tamene, Aklilu Habte, Demelash Woldeyohannes, Habtamu Tamrat, Fitsum Endale, Tekle Eajo, Abel Afework

**Affiliations:** 1 School of Public Health, College of Medicine and Health Sciences, Wachemo University, Hosanna, Ethiopia; 2 Department of Orthopaedic Surgery, School of Medicine, College of Medicine and Health Sciences, Wachemo University, Hosanna, Ethiopia; 3 Dilla University Referral Hospital, Dilla University, Dilla, Ethiopia; The Technical University of Kenya, KENYA

## Abstract

**Background:**

Water is essential for maintaining human life, health, and dignity. Untreated water consumption causes 1.8 million deaths annually, over 99.8% of which happen in developing nations and 90% of which include children. Point-of-use water treatment enables people without reliable access to safe drinking water to reduce contamination and minimize microbial risk levels. This Systematic Review and Meta-analysis was, therefore, used to identify, select, and critically appraise relevant evidence about water treatment practices and their associated factors among Ethiopian households.

**Methods:**

PubMed, Science Direct, Web of Science, Scopus, Embase, Google Scholar, ProQuest, and other databases were searched for studies published before May 5, 2022. The final synthesis included twelve investigations. Microsoft Excel was used to extract the data, and STATA 16 was used for the analysis. The Joanna Briggs Institute’s Critical assessment checklist for prevalence studies was used to evaluate the quality of the included studies. Egger’s test and funnel plot were used to assess publication bias. I^2^ statistics were calculated to check for study heterogeneity. The DerSimonian and Laird random-effects model was used to analyze the pooled effect size, odds ratios, and 95% confidence intervals across studies. Analysis of subgroups was done by publication year and geographic region.

**Results:**

Of the 550 identified articles, 12 studies were eligible for analysis (n = 4849 participants). The pooled prevalence estimate of point-of-use water treatment practice among Ethiopian homes was 36.07% (95% CI: 21.94–50.19, I^2^ = 99.5%). Receiving training from Community health workers (OR, 1.7; 95% CI: 1.33–2.08), female headship (OR, 2.52; 95% CI: 1.60–3.44), and household wealth (OR, 1.6; 95% CI: 1.19–2.16) were significantly associated with point-of-use water treatment practice.

**Conclusion:**

Despite the absence of safely managed water sources, very few homes routinely treated their drinking water. Adoption of water treatment practices necessitates ongoing communication and assistance from health extension personnel. Moreover, program planners must be aware of the many user categories that households may fall under to guarantee that ongoing training messages and treatment products reach every home.

## Introduction

The availability of adequate and clean water is critical to the well-being of a community. Water is thus fundamental to life, health, and human dignity [1]. In addition to the benefits to public health, all people have the right to safe and sufficient access to water for drinking, cooking, and personal and domestic hygiene and because of the potential for serious and widespread outbreaks of waterborne diseases, controlling the risk of microbiological contamination of drinking water is critical [2].

Globally, untreated water consumption accounts for an estimated 1.8 million deaths, with over 99.8% occurring in developing countries and 90% involving children [3]. The sixth Sustainable Development Goal (SDG) seeks to ensure universal access to and sustainable management of water and sanitation. Target 6.1 of the SDGs is specifically designed to achieve the goal of universal and equitable access to safe and affordable drinking water for all by 2030 [4].

Despite progress toward universal coverage of safely managed drinking water, 844 million people around the world continue to lack access to basic water services, and more than 2.1 billion people lack on-premises access to safely managed drinking water [5]. In 2020, the global coverage of safely managed drinking water at home was 74%. This figure was even lower for developing countries, with only 30% of Sub-Saharan Africans and 13% of Ethiopians having access to safely managed water services [6].

Household water treatment (HWT), also known as point-of-use water treatment, provides a means of reducing contamination to lower microbiological risk levels in communities that lack reliable access to safe drinking water by treating water that has been contaminated both at the source and through domestic handling [7]. A large body of evidence proves that when point-of-use water treatment methods are used correctly and consistently, diarrheal diseases can be reduced by as much as 45 percent. HWT, which includes boiling, sedimentation, filtration, chlorination, and solar disinfection (SODIS), is one of the seven strategic areas announced by the World Health Organization (WHO) and the United Nations Children’s Fund (UNICEF) for the prevention of diarrhea and other waterborne diseases through community-wide participation [8–10]. It is also a priority area under Ethiopia’s current national drinking water quality monitoring strategic direction [11].

Studies in Ethiopia show that point-of-use water treatment practice in households ranges from 6% to 76% [11, 12]. The sexual orientation of the household head, level of education, familiarity with water treatment methods, and type of water source were all noted as significant influences on point-of-use water treatment in the primary studies examining domiciliary water treatment practices [13–15]. Although Ethiopia’s water quality levels have been well documented at both the national and international levels, little is known about how and why improvements in point-of-use treatment behavior occur in some households but not others.

The sparse literature on the potential causes of variability and inconsistency in Ethiopian households’ implementation of point-of-use water treatment reveals that we continue to lack viable models for standards and techniques that can work at scale to ensure water safety in situations where risks are prevalent, compliance costs are high, and enforcement capacity is limited. Systematic reviews and Meta-analyses must therefore be used to identify, select, and critically appraise relevant evidence about HWT practices and their associated factors among Ethiopian households. The findings will aid in the consolidation of previous findings and will demonstrate the effects of relevant variables in domiciliary safe water handling. Combining information from multiple data sources can enhance estimates of health-related measures by using one source to supply information that is lacking in another. Furthermore, identifying the antecedents of point-of-use treatment that have the most significant effects vs. those that have less significant effects may assist scholars, practitioners, and policymakers in determining the best course of action.

## Methods and materials

### Review typology

A systematic review was conducted to evaluate and synthesize existing evidence, identify research gaps in the evidence base, and make recommendations. The Preferred Reporting Items for Systematic Reviews and Meta-Analysis (PRISMA) guideline was used for this review and meta-analysis (S1 File). The review protocol has been registered at the international prospective register of systematic reviews (PROSPERO) (ID: CRD42022344695).

### Information sources and search strategies

PubMed/MEDLINE, Google Scholar, African Journal Online (AJOL), Hinari, Science Direct, ProQuest, Directory of Open Access Journals, POPLINE, and Cochrane Library were searched from inception to 2022-05-05 (S2 File). The electronic database search was then supplemented with grey literature found on Google Scholar, Google search, and the Ethiopian University digital repositories (such as the Addis Ababa University Digital Library, and Jimma University Digital Library, Bahir Dar University Digital Library). To ensure a thorough search of the literature, reference lists from included studies were also scanned.

### Eligibility criteria

#### Inclusion criteria

**Table:** 

Study designs:	All types of quantitative observational studies
Study setting:	All documents reporting on point-of-use water treatment practice in Ethiopian households, regardless of their study area.
Time frame:	All studies published up to May 5, 2022, were taken into account.
Publication condition:	Articles published in peer-reviewed journals and relevant grey pieces of literature were included in this review.
Language:	Only articles reported in English were considered.

#### Exclusion criteria

The analysis excluded qualitative studies, reviews, commentaries, letters to the editor, interventional studies, and other opinion publications. The title, abstract, and full text of the articles were analyzed and assessed before they were included in the final review and meta-analysis. Studies that were not accessed after at least two email contacts with the primary authors were removed because assessing methodological quality in the absence of the complete text was difficult. Studies that appeared in multiple search terms, were published in a language other than English, self-identified as pilot/feasibility work, had no new outcome measures, and had salami publications were all excluded.

### Screening

All of the references were imported and de-duplicated using Endnote X9.3.3 (Thompson Reuter, CA, USA). AT and HT screened all references at the title, abstract, and full-text levels, with 20% screened again. The criteria for exclusion were recorded at each stage. In case there was any doubt, a reference was included in the next round of reviews. A third reviewer (DW) independently screened 10% of the removed titles, abstracts, and full texts; the investigators compiled the screened articles, and any disagreements were resolved by consensus.

### Study selection and data extraction

AT and HT used an excel spreadsheet to extract descriptive data, including the first author, type of publication, year of study/publication, objectives, study region, study population (age, sample size, gender); study design, sample size, response rate; the proportion of households with HWT practices, odds ratio, and 95% confidence intervals (CIs).

### Outcome measurement

The primary outcome of this study was point-of-use water treatment practice. It was deemed safe if/when households followed the recommended protocols of the WHO tool kit for household water treatment and storage [16], which included boiling, adding bleach/chlorine, filtering, solar disinfection, and settling water treatment before use. We included research that met the aforementioned criteria.

### Quality appraisal

To assess the quality of included studies and the risks of bias, the Joanna Briggs Institute (JBI) quality assessment tool for prevalence studies was used. Two reviewers independently assessed the quality of the included studies (FE and TI). The 9 parameters of the assessment tool are (1) appropriate sampling frame, (2) proper sampling technique, (3) adequate sample size, (4) study subject and setting description, (5) sufficient data analysis, (6) use of valid measurement for the identified conditions, (7) valid measurement for all participants, (8) using appropriate statistical analysis, and (9) adequate response rate [17].

A score of 1 was assigned if none of the parameters were met. When the information provided was insufficient to assist us in making a decision, we agreed to rate an item as a 1 (failure to satisfy a specific item). Bias risks were classified as low (total score of 0 to 2), moderate (3 or 4), or high (total score, of 5 to 9). Finally, this review included articles with low to moderate bias risk (S3 File).

### Statistical methods and analysis

The data were extracted using a Microsoft Excel spreadsheet, and the statistical analysis was done with STATA™ 16 statistics software. Meta-analysis was run to compute the pooled prevalence of point-of-use water treatment and its determinants. To examine heterogeneity between studies, the I^2^ test was computed and there was significant heterogeneity between the studies (I^2^ = 99%, p<0.001). As a result, Der Simonian and Laird’s random-effects model was used to calculate the pooled effect. Subgroup analyses were conducted by region and year of study. Accordingly, the pooled prevalence of point-of-use water treatment practice across subgroups and their corresponding 95% CI were presented using forest plots.

A *p*-value of 0.372 and 0.161 for Begg’s and Egger’s tests, respectively, implied that a small-study effect was less likely (P<0.05 is considered statistically significant). Using a random-effects meta-analysis, the results of the included studies were pooled, and they were presented as percentages of point-of-use water treatment practices and associated factors with 95% confidence intervals. The influence of a single study on the overall pooled estimate was investigated using a sensitivity analysis with a random-effects model. The meta-regression was used to ascertain the most likely cause of heterogeneity and used sample size (p-value of 0.8575) and year of publication (p-value of 0.3184) as input parameters. Statistical significance is marked at the p<0.05 level

## Results

### Description of included studies

**Fig 1:**
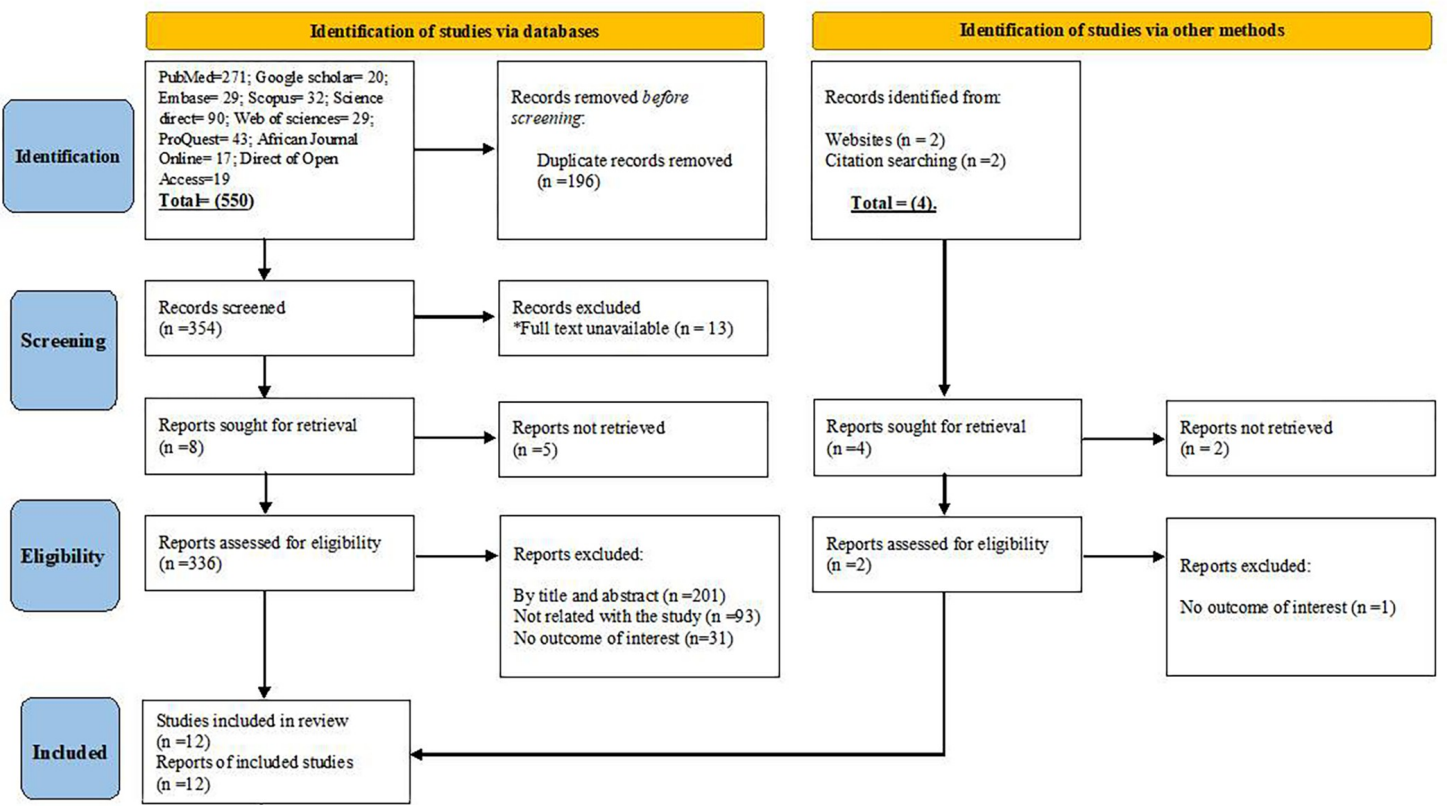
A PRISMA flowchart for the selection of eligible studies on point-of-use water treatment practices among Ethiopian households.

PubMed (n = 271), Google Scholar (20), African Journal Online (17), Embase (n = 29), Science Direct (n = 90), ProQuest (n = 43), Direct of Open Access Journals (19), Web of Sciences (n = 29), and other sources (n = 4) together yielded a total of 550 articles. We screened the titles and abstracts of 384 publications, removing 169 duplicates. Finally, 12 satisfied the inclusion requirements and were included in the analysis. Six of the included studies came from PubMed, two from Embase, two from Google Scholar, one from the Directory of Open Access Journals, and the final study came from citation searching (Fig 1).

### Characteristics of included studies

Table 1 demonstrates that 12 studies satisfied the requirements for inclusion. All of the included studies were cross-sectional by design. The total number of households included in the current systematic review and meta-analysis was 7158. The median response rate for the included investigations was 98.5%. The studies were conducted between 2016 through 2022. Sample sizes in the included studies ranged from 377 to 865.

**Table 1:** Baseline characteristics of the included studies, 2016–2022.

Authors’ Name& Publication Year	Study Area	Study Region	Study Setting	Sampling	Sample Size	RR	POU water treatment	Risk of bias
Birara et al., 2018 [18]	Bahirdar City	Amhara	Urban	Srs	418	98.6%	76.3	Low
Tunje et al., 2019 [14]	Chencha district^+^	SNNPR	Rural	SRS	739	100%	65.5	Moderate
Tafesse et al., 2021 [19]	Gibe district^+^	SNNPR	Rural	SRS	627	94.4%	34.3	Low
Admasie et al., 2022 [20]	Sodo zurya district^+^	SNNPR	Rural	Srs	836	100%	44.1	Low
W/tsadik et al., 2022 [12]	Bure Town	SNNPR	Urban	SRS	418	98.9%	29.9	Low
Azage et al., 2018 [21]	Baso Liben	Amhara	Rural	SRS	865	98.8%	26	Moderate
	Yilmana Densa							
	Fogera districts^+^							
Bitew et al., 2017 [22]	Dabat district^+^	Amhara	Rural	SRS	845	98.7%	23.1	Moderate
Belay et al., 2015 [23]	Burie Zuria^+^	Amhara	Rural& Urban	SRS	797	98.2%	44.8	Low
Merga et al., 2021 [11]	Assosa district^+^	Benishangul Gumuz	Rural& Urban	SRS	378	95.17%	13.2	Low
Geremew et al., 2018 [13]	Eastern hararge and Kersa zones*	Oromia and Harari	Rural& semi-urban	SRS	377	100%	31	Low
Tsegaye et al., 2020 [15]	Degadamot district^+^	Amhara	Rural	SRS	845	100%	14	Low
Eticha et al., 2022 [7]	Ameya district^+^	Oromia	Urban and rural	SRS	413	100%	30	Low

RR: Response rate.

SRS: Systematic random sampling.

Srs: simple random sampling.

SNNPR: Southern Nations Nationalities and Peoples’ Region.

*The second-level administrative divisions of Ethiopia after Regional governments.

^+^ The third-level administrative divisions of Ethiopia after Regional governments and zones.

The 12 studies were conducted across four regions. Four investigations were conducted in the South Nations and Nationalities and Peoples’ Region (SNNPR), two in the Oromia region, five in Amhara, and one in the Benishangul Gumuz regional state. While the majority of the studies gathered their data using questionnaires and interviews, three also used inspection to look into the cleanliness and sanitary conditions of the water storage containers. The majority (75%) of the studies had a low risk of bias. Additionally, further analysis was performed on all investigations to identify contributing factors to point-of-use water treatment (Table 1).

### Meta-analysis of pooled prevalence of point-of-use water treatment practice

**Fig 2:**
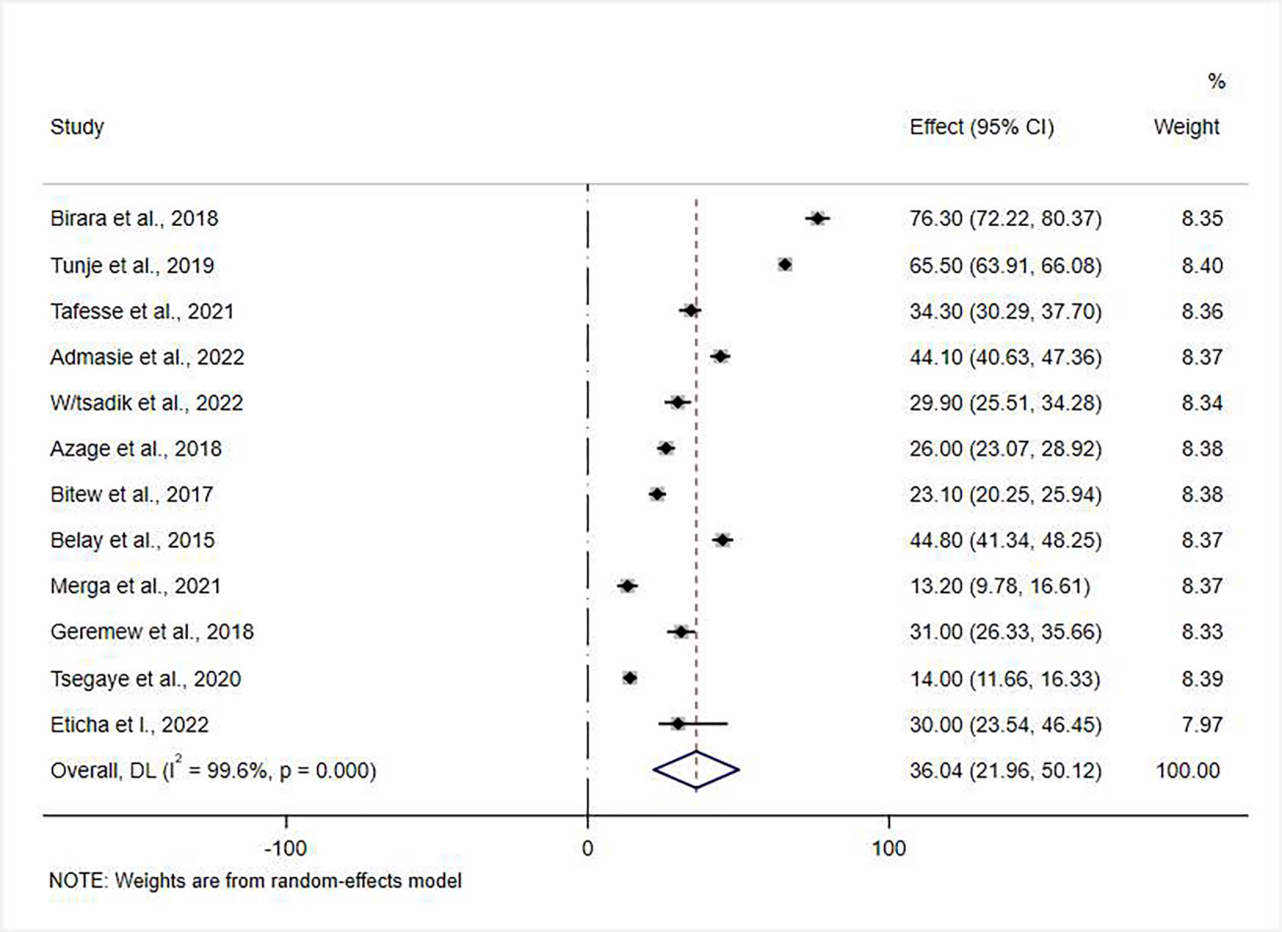
Forest plot depicting pooled prevalence estimate of POU water treatment practices among Ethiopian households. NOTE: Weights are from random-effects model.

There was significant heterogeneity in the prevalence estimate among studies (P < 0.001; I^2^ = 99.5%). We, therefore, used a random effect model. Based on the DerSimonian-Laird random-effects model, the pooled prevalence estimate of point-of-use water treatment practice among Ethiopian homes was 36.07% (95% CI: 21.94–50.19). A forest plot depicts the prevalence estimates of POU water treatment practice among households (Fig 2).

### Point-of-use water treatment by type among users

Table 2 further displays the general frequency of various point-of-use treatment alternatives. In the present Meta-analysis, filtration was the approach that 45.04% of all households used (95% CI 26.9–59.16). Boiling accounted for 44.59% (95% CI 27.04–62.15). Chemical POU was used in 38.53% of the households (95% CI 19.33–67.72). None of these three methods can however be statistically distinguished from one another. On the other hand, all three were used significantly more than the settling method, with only 9.74% of the households picking the stand and settle technique as their chosen method of treatment (95% CI1.73–17.84) (Table 2).

**Table 2:** Overall pooled prevalence of point-of-use water treatment by type and preferences, among Ethiopian households.

POU	Pooled effect		
Treatment technology	Sample size	POU treatment	Prevalence (95%CI)
**Boiling**	2949	1319	44.59 (27.04–62.15)
Tafesse et al., 2021 [19]			
Admasie et al., 2022 [20]			
W/tsadik et al., 2022 [12]			
Bitew et al., 2017 [22]			
Belay et al., 2015 [23]			
**Chemical** ^**+**^	3985	1443	38.53 (19.33–67.72)
Tafesse et al., 2021 [19]			
Admasie et al., 2022 [20]			
W/tsadik et al., 2022 [12]			
Belay et al., 2015 [23]			
Tsegaye et al., 2020 [15]			
Birara et al., 2018 [18]			
**Settling**	2527	228	9.74 (1.73–17.84)
Admasie et al., 2022 [20]			
Belay et al., 2015 [23]			
Bitew et al., 2017 [22]			
**Filtration** *	1463	659	45.04 (26.9–59.16)
W/tsadik et al., 2022 [12]			
Birara et al., 2018 [18]			
Tafesse et al., 2021 [19]			

Note: The prevalence is higher than 100 because some families coupled water treatment technologies.

^**+**^ adding chlorine, adding bleach.

*cloth filtration, sand filtration, gravel filtration, ceramic filter.

### Subgroup analysis

**Fig 3:**
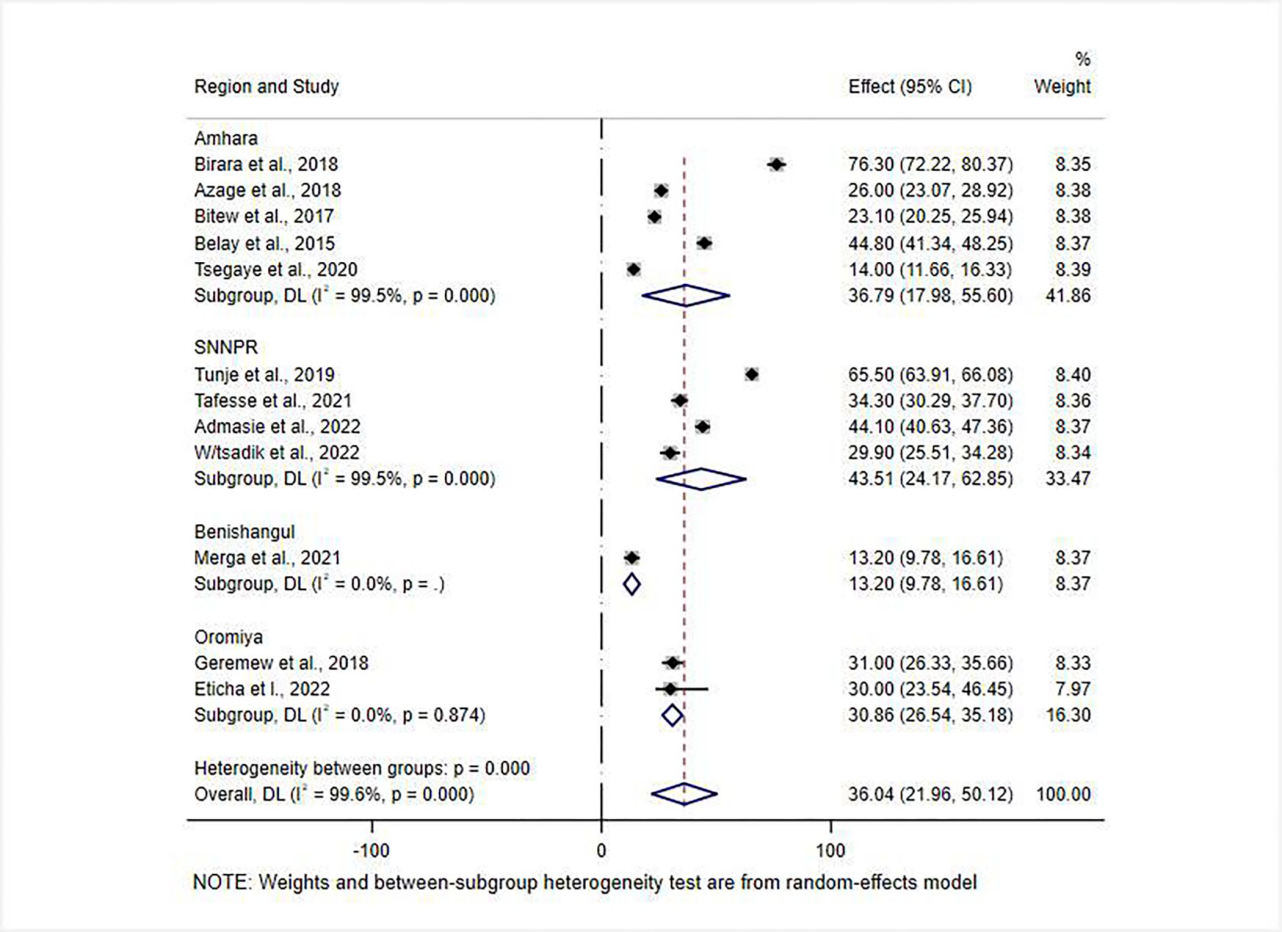
Sub-group analysis by region for the pooled prevalence of POU water treatment practices among Ethiopian households. NOTE: Weights and between-subgroup heterogeneity test are from random-effects model.

A subgroup analysis based on geographical location (country region) was undertaken to see if there were any regional differences in water treatment practices. As a result, the SNNPR and Benishangul Gumuz regions had the highest and lowest prevalence of POU water treatment at 43.51% (95% CI: 24.17–62.85) and 13.20% (95% CI: 9.78–16.61) respectively (Fig 3).

### Heterogeneity and publication bias

**Fig 4:**
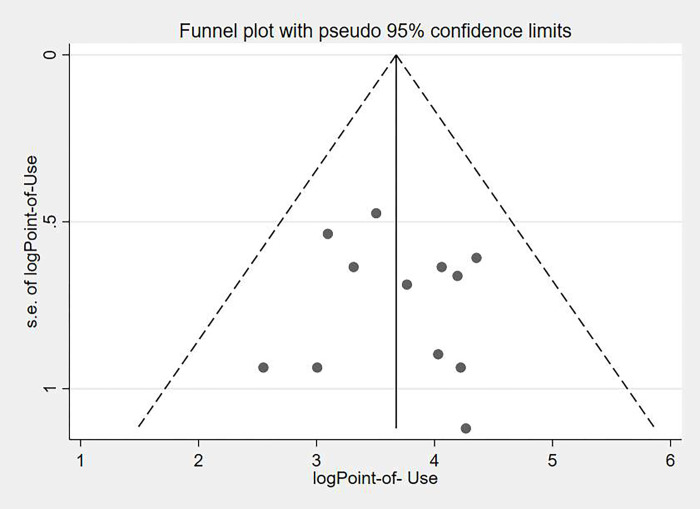
Funnel plot displaying publication bias of studies reporting POU water treatment practices among Ethiopian households.

Visual inspection of the symmetrical funnel plot (Fig 4) revealed no publication bias, which was statistically supported by Begg’s test (P = 0.372) and Egger’s test (bias coefficient (B) = 17.6 (95% CI = − 35.62–0.369; P = 0.16)).

A statistical heterogeneity test via a quantitative technique of univariate meta-regression was also conducted by taking publication year and sample size into consideration. The findings demonstrate that neither of the two significantly affected the study’s heterogeneity (Table 3).

**Table 3:** Univariate meta-regression of factors related to the heterogeneity of POU water treatment practices among Ethiopian households, 2022.

Variables	Coefficient	P-value
Sample size	-2.697784	0.8575
Year	-.00511409	0.3184

### Sensitivity analysis

**Fig 5:**
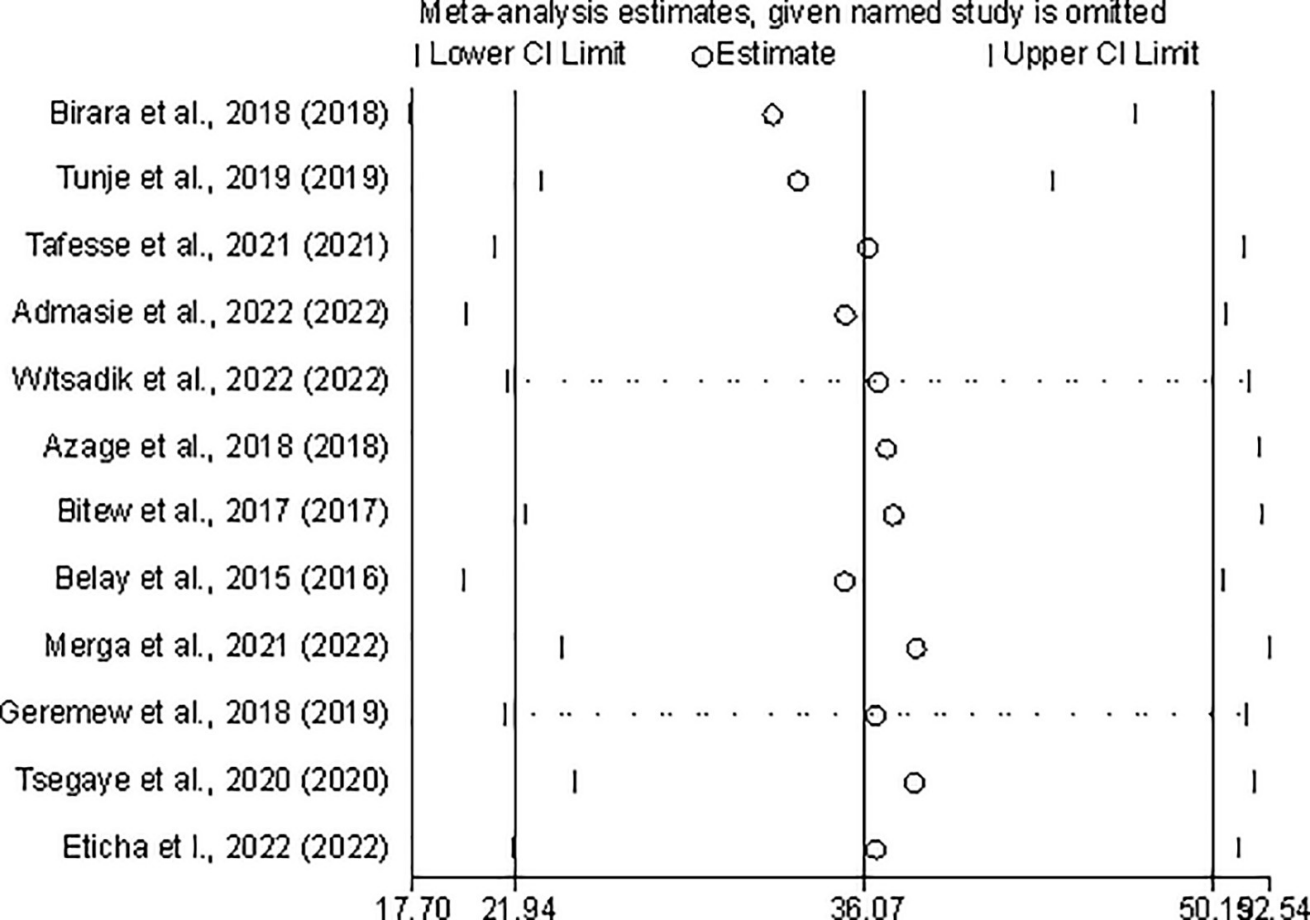
Sensitivity analysis for estimates of POU water treatment practices among Ethiopian households (number of estimates = 12).

Sensitivity analysis using the random-effects model revealed that no single study influenced the overall prevalence of POU water treatment practices among Ethiopian households (Fig 5).

### Factors associated with point-of-use water treatment

**Fig 6:**
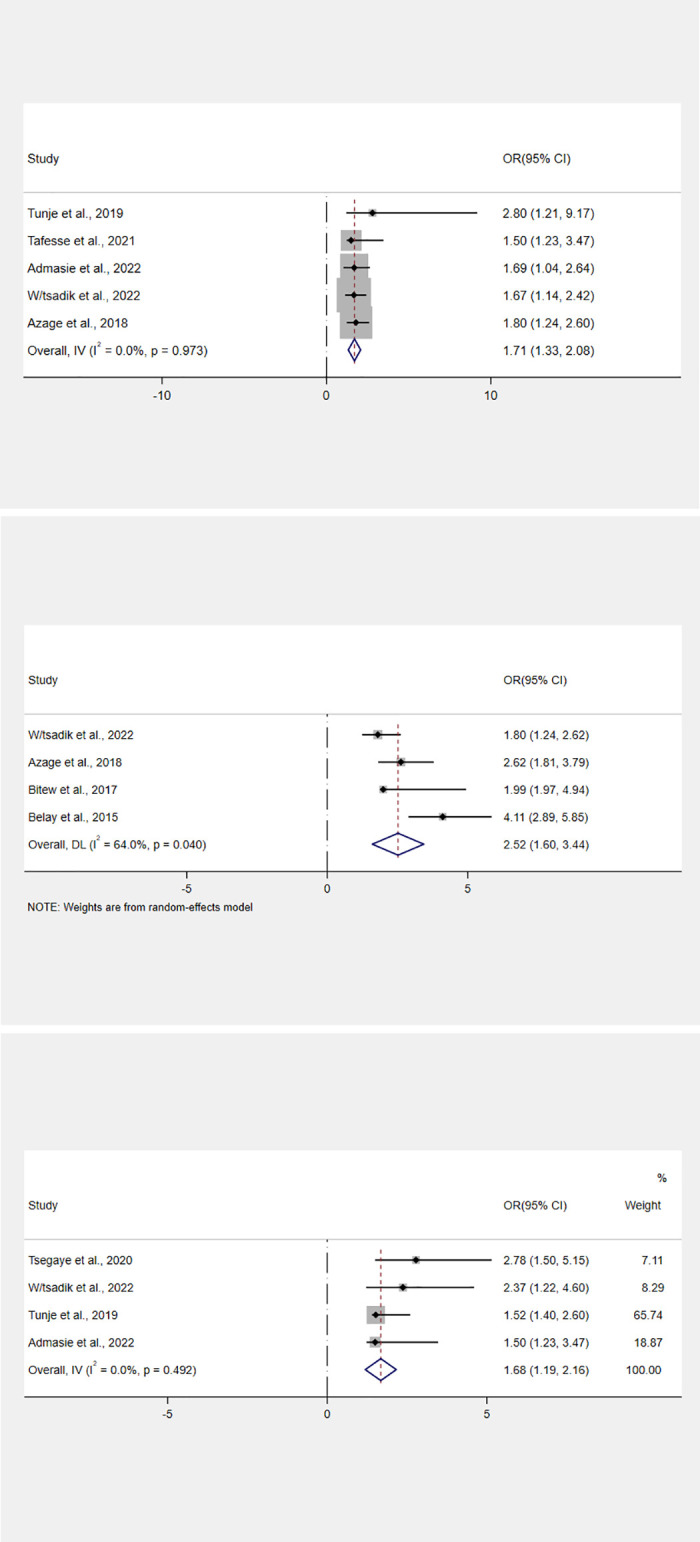
(a) Forest plot of the association between training and point-of-use water treatment practices in Ethiopia. (b) Forest plot of the association between the sexual identity of household head and point-of-use water treatment practices in Ethiopia. (c) Forest plot of the association between wealth index and point-of-use water treatment practices in Ethiopia. NOTE: Weights are from random-effects model.

The meta-analysis of factors related to point-of-use water treatment practices included nine studies involving six factors (S4 File). Community health workers’ training increased a household’s likelihood of treating its water by 1.7 times compared to those who did not receive training (OR, 1.7; 95% CI: 1.33–2.08) (Fig 6A).

Similarly, households headed by women were 2.5 times more likely than those headed by men to practice water treatment at the point of use. (OR, 2.52; 95% CI: 1.60–3.44) (Fig 6B).

Additionally, the practice of point-of-use water treatment was associated with household affluence. In comparison to households in the lowest wealth quintiles, households in the upper wealth quintile were 1.6 times more likely to practice HWT. (OR, 1.6; 95% CI: 1.19–2.16) (Fig 6C).

## Discussion

Safe drinking water is a basic need and a human right that should be available to everyone. Securing access for all would significantly reduce diseases and death, particularly among children. Centralized water treatment is the most popular (and long-term) solution to the issue, but it is expensive, time-consuming, and will take decades to implement. While improvements are being made to access, other strategies, such as point-of-use water treatment, are needed to meet urgent demands [24]. Therefore, the current findings offer pertinent insight into the POU water treatment practice in Ethiopian households, as well as its geographic distribution and underlying factors.

In the present meta-analysis, point-of-use water treatment methods were used by 36.07% (95% CI: 21.94–50.19) of households in Ethiopia. This pooled proportion was lower when compared to a study done in Uganda at 76% (95% CI: 61.94–80.19) [25], Notwithstanding the lack of systematic reviews or meta-analyses on similar topics in Ethiopia or elsewhere, this shows that there are significant disparities in POU water treatment practices between countries, which may be caused by social, cultural, and/or environmental factors. Moreover, because Ethiopia is one of the least developed countries in the world; issues including population growth, a lack of funding for health initiatives, and a lack of a reliable mechanism to track setbacks in improving household water treatment practices also play a significant role [12]. Ethiopia also imports many chemical-based additives and filters. POU treatment products, however, have not always been regarded as a standard medical intervention that qualifies for tax exemption, despite being supported by national health plans. End consumers are responsible for paying the taxes on these goods, which drives up the product price [23]. And in the context of widespread poverty, the lack of access to affordable products may constitute the primary roadblock to continued use for many.

Numerous technologies have been introduced and incorporated into POU treatment systems over the past ten years [26, 27]. In the current Meta-analysis, while some families used a multi-barrier approach to treating water (coupling multiple treatment options to reduce the risk of infection), others used a single method for water treatment. While more research is required to understand the inhibitors and motivations influencing preferences for water treatment types, very little is up for debate regarding the provision of affordable, scalable, and efficient solutions to the significant challenge of providing potable drinking water in lower-income settings.

In many cases, the effects of handling water in the home can be linked to a variety of complex, dynamic relationships between people and their environment [7, 13]. This meta-analysis identified considerable regional variations in point-of-use water treatment practices. The SNNPR 43.51% (95% CI 17.98–55.60) and Benishangul-Gumuz 13.20% (95% CI 9.78–16.61) regions had the highest and lowest rates of point-of-use water treatment, respectively. The Ethiopian government has categorized the Benishangul-Gumuz region as a developing regional state as a result of the high prevalence of poverty and socioeconomic indices that are far below national standards [28]. While differences in socio-demographic traits and the number of studies included in each category of analysis may account for variations in POU water treatment rates between regions, it is known that inadequacies, inconsistencies, inequities, and inefficiencies pose some of the biggest threats to water safety in the home [29].

In the present Meta-analysis, community health workers’ training increased a household’s likelihood of treating its water compared to those who did not receive training. Community health workers promote the correct use of POU treatment products in each village. The major goals of promotion are to fuel demand for treatment products and facilitate long-term use [12]. The expansion of HEWs to offer more services related to curative therapy, meanwhile, may prevent HEWs from offering such training. Health educators may not have enough time to support water treatment behaviors and urge community members of various user kinds and preparedness levels to treat their water [30]. Therefore, moving forward, community training programs should involve a variety of community-based stakeholders, such as civil societies (CSO), to take into account the change in duties of HEWs. Local CSOs, after all, have a strong reputation for being the voice of the community and have the ability to affect public opinion.

Female-headed homes in Africa are, on average, poorer than male-headed households, but studies also show that the children from these households fare better. On long-term measures of nutritional status, children in households with female heads perform much better [31]. According to studies, women are more competent than male leaders to divide income and resources among family members evenly and spend more on the health of family members [32]. Similar to this, in the current meta-analysis, households headed by females were 2.5 times more likely to practice water treatment at the point of use than those led by men. On the other hand, even though they are often the primary implementers of household practices, most women in male-headed homes do not have a voice equal to their partners in household spending [33]. Thus, in the context of pervasive poverty, access to money or treatment products among primary caregivers appears to be a crucial element for sustainable water treatment.

Additionally, the practice of point-of-use water treatment was associated with household affluence. In comparison to households in the lowest wealth quintiles, households in the upper wealth quintile were more likely to practice HWT. POU treatment systems should ideally be simple to use, inexpensive, and low maintenance. But just because something is inexpensive doesn’t imply everyone can afford it [7]. Solar disinfection is an inexpensive and simple technique; however, the price of plastic bottles may be a major factor in whether or not it is accepted in communities. Boiling water is one of the most common methods, however, doing so necessitates the purchase of cooking fuel and boiling pots [34]. Efforts to reduce the price of such items and their associated costs could be successful given that the cost of water treatment products has been frequently highlighted as a barrier to continuous use. Despite Ethiopia’s policy of prohibiting public subsidies for household water treatment products, evidence demonstrates that public subsidies have been successful in other nations [35, 36].

This analysis has several limitations. First, the search was limited to English-language articles exclusively. Second, all of the investigations were observational, and the results were not supported by qualitative methods. Finally, this meta-analysis only included four regions of Ethiopia.

### Future scope

It is difficult to collect data on the number of individuals who become ill from diseases that could be brought on by improper domestic water management practices. However, attempts should be made to gather this information wherever possible. Furthermore, a review of the costs related to WBDs should be done to inform managers and policymakers. Future research should also consider the microbiological risk levels in areas without reliable access to clean drinking water. Furthermore, additional research should define precise study boundaries, such as urban or rural, to thoroughly examine ecological, hydrological, and resource variables and implement customized interventions. More research should also concentrate on Ethiopia’s emerging regions since the drawbacks of achieving optimal point-of-use water treatment have not been adequately articulated. To paint a holistic picture of the situation, interdisciplinary and systemic research encompassing both qualitative and quantitative designs should also be prioritized over traditional disciplinary research. Finally, even though the current study recognized training as a crucial factor in determining POU water treatment practice, it did so within the framework of a broader picture. Future studies may need to further dissect it, looking at the nature and frequency of training as well as the information presented.

## Conclusion

This Meta-analysis contributes to our understanding of the factors affecting the adoption and continued use of technologies for household water treatment. Our analysis demonstrates that very few households continuously treat their drinking water, despite the limited availability of safely managed water sources. Adoption of water treatment methods necessitates constant communication and cooperation from all parties; In addition to the ongoing clean water education that Community health workers provide, future programs should stress this. For instance, community mobilization initiatives that encourage water treatment through the involvement of local leaders, women’s organizations, etc. may increase potential adoption.

After combining information from multiple data sources on POU water treatment practices, the present meta-analysis identified training from Community health workers, household affluence, and female headship as significant drivers of point-of-use water treatment in the Ethiopian context. Program planners should be aware of such user categories that homes may fall under to guarantee that varied user types are taken into account in intervention activities.

## Supporting information

S1 File PRISMA checklist. (DOC)

S2 File Literature search strategies. (DOCX)

S3 File Quality appraisal checklist. (DOCX)

S4 File Extracted data. (XLSX)

## List of abbreviations

AOR: Adjusted odds ratio
CI: Confidence Interval
HWT: Household water treatment
JBI: The Joanna Briggs Institute
LMICs: low- and middle-income countries
OR: Odds Ratio
PRISMA: Preferred Reporting Items for Systematic Reviews and Meta-Analyses
POU: Point-of-Use
SNNPR: Southern Nations Nationalities and Peoples’ Region
WBDs: waterborne diseases
