# Supplementary material for: Water treatment at the point-of-use and treatment preferences among households in Ethiopia: A contemporaneous systematic review and meta-analysis
Source: PLoS One. 2022 Oct 27;17(10):e0276186. doi: 10.1371/journal.pone.0276186 (PMC9612552; doi:10.1371/journal.pone.0276186)
Supplement: S2 File — (DOCX) [file pone.0276186.s002.docx]

Supplementary file 2: Table S2. Literature Search strategies for point-of-use water treatment and associated factors among Ethiopian Households, May 05, 2022

| S.N. | Database | Search strategy | Search results |
| --- | --- | --- | --- |
| 1 | PubMed | ((("point-of-use"[MeSH Terms] OR "household"[All Fields] OR "households"[All Fields] OR "domicillary"[All Fields]) AND "water purification"[MeSH Terms] OR ("water"[All Fields] AND "purification"[All Fields]) OR "water purification"[All Fields] OR ("water"[All Fields] AND "treatment"[All Fields]) OR "water treatment"[All Fields] AND "Practice"[All Fields] AND "Associated factors"[All Fields]) OR "related factors"[All Fields] OR "Determining factors"[All Fields]) AND ("Ethiopia"[MeSH Terms] OR "Ethiopia"[All Fields] OR "Ethiopia’s"[All Fields]) | 271 |
| 2 | Google scholar | allintitle: Ethiopia "water treatment practice" OR "Household water treatment practice" OR " Point-of-use water treatment practice" OR "household water handling practice" OR "small-scale water treatment practice" OR "water purification practice" | 20 |
| 3 | Embase | ('water treatment' OR (('point-of-use'/exp OR point-of-use) AND water treatment)) AND ('ethiopia'/exp OR ethiopia) | 29 |
| 4 | Scopus | TITLE-ABS-KEY ( ''water treatment AND households'' AND ethiopia ) | 32 |
| 6 | Science direct | Find articles with these terms: water treatment and Ethiopia(Research articles highlighted)= | 90 |
| 7 | Web of sciences | TOPIC: (water treatment and Ethiopia) | 29 |
| 8 | ProQuest | ab(water treatment) AND ab(Ethiopia) | 43 |
| 9 | African Journal Online | “water treatment" AND "Ethiopia” | 17 |
| 10 | Directory of Open Access Journals | “water treatment" AND "Ethiopia” | 19 |
|  | Total |  | 550 |
