# Supplementary material for: Water treatment at the point-of-use and treatment preferences among households in Ethiopia: A contemporaneous systematic review and meta-analysis
Source: PLoS One. 2022 Oct 27;17(10):e0276186. doi: 10.1371/journal.pone.0276186 (PMC9612552; doi:10.1371/journal.pone.0276186)
Supplement: S3 File — (DOCX) [file pone.0276186.s003.docx]

**Supplementary file 3**

**Table S3: Individual quality assessment of articles included in the review, studies published between 2016 and 2022.**

| **Authors, Year** | **Sampling frame** | **Sampling strategy** | **Sample size** | **Description of research setting & population** | **Data analysis conducted with sufficient coverage** | **Valid methods used for the identification of the condition** | **Reliability of the instrument used** | **Statistical analysis methods** | **Response rate** | **Total** | **Risk of bias** |
| --- | --- | --- | --- | --- | --- | --- | --- | --- | --- | --- | --- |
| Birara et al., 2018 | 0 | 0 | 0 | 0 | 0 | 1 | 1 | 0 | 0 | 2 | Low |
| Tunje et al., 2019 | 1 | 0 | 0 | 0 | 0 | 1 | 1 | 1 | 0 | 4 | Moderate |
| Tafesse et al., 2021 | 0 | 0 | 0 | 0 | 0 | 1 | 1 | 0 | 0 | 2 | Low |
| Admasie et al., 2022 | 0 | 0 | 0 | 0 | 0 | 1 | 1 | 0 | 0 | 2 | Low |
| W/tsadik et al., 2022 | 0 | 0 | 0 | 0 | 0 | 1 | 1 | 0 | 0 | 2 | Low |
| Azage et al., 2018 | 0 | 0 | 0 | 0 | 0 | 1 | 1 | 1 | 0 | 3 | Moderate |
| Bitew et al., 2017 | 1 | 1 | 0 | 0 | 0 | 1 | 1 | 0 | 0 | 4 | Moderate |
| Belay et al., 2015 | 0 | 0 | 0 | 0 | 0 | 0 | 0 | 0 | 0 | 0 | Low |
| Merga et al., 2021 | 0 | 0 | 0 | 0 | 0 | 1 | 1 | 0 | 0 | 2 | Low |
| Geremew et al., 2018 | 0 | 0 | 0 | 0 | 0 | 1 | 1 | 0 | 0 | 2 | Low |
| Tsegaye et al., 2020 | 0 | 0 | 0 | 0 | 0 | 1 | 1 | 0 | 0 | 2 | Low |
| Birara et al., 2018 | 0 | 0 | 0 | 0 | 0 | 1 | 1 | 0 | 0 | 2 | Low |
| Tunje et al., 2019 | 0 | 0 | 0 | 0 | 0 | 1 | 1 | 0 | 0 | 2 | Low |
